# Validation of the Italian version of the Revised Prenatal Coping Inventory (NuPCI) and its correlations with pregnancy-specific stress

**DOI:** 10.1186/s12884-020-03159-5

**Published:** 2020-08-14

**Authors:** Chiara Penengo, Chiara Colli, Marco Garzitto, Lorenza Driul, Maddalena Cesco, Matteo Balestrieri

**Affiliations:** 1 Unit of Psychiatry, Department of Medicine (DAME), University of Udine, Udine, Italy; 2 Clinic of Obstetrics and Gynecology, Department of Medicine (DAME), University of Udine, Udine, Italy

**Keywords:** Pregnancy, Stress, Coping, Validation, NuPCI, NuPDQ

## Abstract

**Background:**

Pregnancy is a period of happiness but also of physical and psychological changes that can lead to distress. Functional coping strategies can reduce the pregnancy specific-stress. This study aimed to assess the psychometric properties of the Revised Prenatal Coping Inventory (NuPCI) in an Italian sample and to investigate how coping strategies were associated with pregnancy-specific stress.

**Methods:**

In this cross-sectional study, low-risk pregnant women (*N* = 211) were assessed with NuPCI, NuPDQ (Revised-Prenatal Distress Questionnaire), Brief-COPE (Coping Orientation to the Problems Experienced), and STAI (State-Trait Anxiety Inventory). The reliability of NuPCI was evaluated by assessing its internal consistency and factor structure (with a Confirmatory Factor Analysis, CFA). The concurrent validity between NuPCI and Brief-COPE and NuPDQ and STAI was investigated. Lastly, the relationship between NuPCI and NuPDQ was analyzed, as well as the ability of these scales to predict Apgar score at birth.

**Results:**

Internal consistency of NuPCI scales was good for Planning-Preparation (ɑ_C_=0.84) and Spiritual-Positive Coping (ɑ_C_=0.81) scales, acceptable for Avoidance (ɑ_C_=0.76) scale. Moreover, the original three-factor structure was confirmed using a CFA with 29 of the 32 items (χ^2^_374_ = 618.06; RMSEA = 0.056, 95% confidence interval: [0.048, 0.063]); CFI = 0.920; and TLI = 0.913). Statistically significant correlations between NuPCI scales and Brief-COPE subscales ranged between *r* = + 0.217 and *r *= + 0.624; also, NuPDQ score was positively correlated with STAI scales (State scale: *r* = + 0.539; Trait scale: *r* = + 0.462). Concurrent validity was confirmed reporting that NuPDQ score was predicted by NuPCI scores (R^2^ = 0.423, *p* < 0.001), positively by Avoidance (β=+0.572) and Planning-Preparation (β=+0.215) and negatively by Spiritual-Positive Coping (β=-0.132). Finally, considering the stress, the effect of the Avoidance and Spiritual-Positive Coping scores respectively in decreasing (+ 155%) and increasing (+ 16%) the Apgar score became stronger.

**Conclusions:**

Italian NuPCI has sound psychometric properties and it is a useful coping measure. NuPDQ showed also a good validity. Our results may suggest a significant role for coping strategies, particularly in modulating the condition of the newborn at birth.

## Background

Pregnancy is a period of joy and happiness, but also a time of physiological, social and emotional changes, which can lead to depression, anxiety, and stress. During gestation, the stress can be mitigated by functional coping strategies, letting women adopt useful responses at emotional, cognitive, and behavioral levels [1].

Coping is defined as any cognitive or behavioral attempt to manage situations that are perceived as stressful [2] because they tax or exceed one’s resources [3]. Previous studies have distinguished two main coping styles, one focused on problems and another on emotions [4]. Further conceptualization distinguishes approach coping (i.e., based on active problem-solving) and avoidance coping (i.e., involving the attempt to avoid or withdraw from stressful situations) [5]. According to Carver, avoidance coping may be interpreted as a form of emotion-focused coping, because it involves the denial of feelings associated with the stressor [6].

Many studies have investigated coping styles during pregnancy, but in most cases, generic coping measures have been used [7]. According to Guardino and Dunkel Schetter [1], the most reliable instruments to evaluate coping in the general population are Folkman & Lazarus’s Ways Of Coping (WOC) [8], and Carver’s Coping Orientation to the Problems Experienced (COPE) [9], also in its short version (Brief-COPE) [6]. However, the Prenatal Coping Inventory (PCI), developed by Yali and Lobel [10], and its revised version, the NuPCI [11], are the specific measures for pregnancy to be preferred. In particular, the good internal consistency of NuPCI has been confirmed in several studies in USA and elsewhere in high and low risk pregnant women [12–16].

The use of appropriate strategies of coping for stressors may help minimize pregnancy-specific stress (PSS). PSS arises from pregnancy-specific issues, like physical symptoms, changes in body image, anxiety about deliver and concerns about the new role in the family [11]. The exposure to chronic stressful conditions can be detrimental to the health of the mother and the unborn child [17]. Convergent evidence suggests that a high level of maternal stress during pregnancy is associated with negative outcomes in the mothers and the newborns, such as low birth-weight, preterm delivery, pre-eclampsia, gestational diabetes, and unplanned caesarean section [12, 18]. Furthermore, pregnancy-specific stress exerts negative effects on offspring regardless of the obstetric risk, suggesting that stress consequences could be partially independent by medical conditions during pregnancy [7, 19]. Infant Apgar score is the most widely used measure of newborn conditions. The Apgar score includes the heart rate, respiratory effort, muscle tone, reflex irritability, and skin color. Scores of 7 to 10 are considered reassuring. The Apgar score was previously used as a measure of neonatal outcome in relation to PSS [20–22], although with inconsistent evidences [23, 24].

Although PSS tends to co-occur with general stress, the distress experienced during gestation is a stronger predictor of altered neurodevelopment in the offspring [25].

A broad range of psychometric instruments has been applied to assess PSS. The following instruments have been identified as the best currently available measures for the different dimensions of PSS: State-Trait Anxiety Inventory (STAI), Edinburgh Postnatal Depression Scale (EPDS), Perceived Stress Scale (PSS), Abbreviated Scale for the Assessment of Psychosocial Status in Pregnancy, Prenatal Life Events Scale, Prenatal Distress Questionnaire (PDQ) [26]. The original PDQ was found to be significantly associated with general stress measures (STAI, Life Event Stress, and PSS) [18]. The Revised Prenatal Distress Questionnaire (NuPDQ) is a particularly appropriate tool to assess PSS; it has shown to have good validity and reliability [7, 16].

Due to the absence of specific instruments to investigate different ways of coping with stress among Italian pregnant women, the main aim of this research was to adapt the NuPCI to the Italian population, evaluating the psychometric properties of the instrument and its construct validity. A second purpose was to study how coping strategies are associated with levels of stress during pregnancy in an Italian sample. Thus, a cross-sectional study was carried out to evaluate the reliability and validity of the NuPCI in different pregnancy periods.

## Methods

### Participants

The study was performed between December 2017 and May 2019, and was proposed to 266 pregnant women receiving prenatal care as outpatients at the Gynecology Clinic of the University Hospital of Udine (Italy): 55 women (20.7%) refused to participate and the final sample comprised 211 participants. The sample size of this study was determined according to Mundfrom et al. [27] that reported a minimum sample size for conducting factor analysis of three to ten times the number of variables (see also: [28]).

Inclusion criteria were age over 18 years, Italian fluency, and absence of pregnancy complications (as routinely evaluated during gynecological visits) until the research assessment. Of the 211 eligible women, 70 were in the first trimester (1–13 weeks), 71 were in the second trimester (14–25 weeks), and 70 were in the third trimester (26–40 weeks).

Socio-demographic and general characteristics of the sample are detailed in Table 1.

**Table 1:** Socio-demographic and general characteristics of the total sample and by-trimester groups, together with scores in coping and stress tests

		**Total sample**	**1**^**st**^ **trimester**	**2**^**nd**^ **trimester**	**3**^**rd**^ **trimester**
**Participants**		**211**	**70**	**71**	**70**
**Age**		32.91 (4.95)	33.07 (4.99)	33.13 (5.28)	32.54 (4.58)
**Previous pregnancy**	0	105 (49.8%)	33 (47.1%)	38 (53.5%)	34 (51.4%)
	1	65 (30.8%)	27 (38.6%)	15 (21.1%)	23 (32.9%)
	2	31 (14.7%)	9 (12.9%)	12 (16.9%)	10 (14.3%)
	3–5	10 (4.7%)	1 (1.4%)	6 (8.5%)	3 (4.3%)
Level of education*	University	101 (47.9%)	32 (45.7%)	33 (46.5%)	36 (51.4%)
	High school	89 (42.2%)	33 (47.1%)	30 (42.3%)	26 (37.1%)
	Middle-school	19 (9.0%)	4 (5.7%)	7 (9.9%)	8 (11.4%)
	Primary school	1 (0.5%)	1 (1.4%)	-	-
**Employment status**	Employed	138 (65.4%)	49 (70%)	42 (59.2%)	47 (67.1%)
	Self Employed	20 (9.5%)	5 (7.1%)	10 (14.1%)	5 (7.1%)
	Unemployed	53 (25.1%)	16 (22.9%)	19 (26.8%)	18 (25.7%)
**Marital status**	Married	137 (64.9%)	45 (64.3%)	47 (66.2%)	45 (64.3%)
	Unmarried	72 (34.1%)	24 (34.3%)	23 (32.4%)	25 (35.7%)
	Divorced	2 (0.9%)	1 (1.4%)	1 (1.4%)	-
**Brief-COPE**	Adaptive coping	2.69 (0.43)	2.66 (0.48)	2.72 (0.44)	2.70 (0.38)
	Maladaptive coping	1.89 (0.32)	1.87 (0.31)	1.93 (0.31)	1.87 (0.33)
	Self-distraction	2.52 (0.71)	2.48 (0.72)	2.58 (0.74)	2.49 (0.68)
	Active coping	3.18 (0.71)	3.22 (0.64)	3.15 (0.80)	3.18 (0.69)
	Denial	1.36 (0.52)	1.44 (0.60)	1.35 (0.52)	1.29 (0.44)
	Substance use	1.04 (0.20)	1.03 (0.15)	1.06 (0.29)	1.02 (0.13)
	Use of emotional support	2.66 (0.82)	2.61 (0.86)	2.65 (0.78)	2.70 (0.83)
	Use of instrumental support	2.76 (0.76)	2.71 (0.84)	2.80 (0.69)	2.76 (0.74)
	Behavioral disengagement	1.45 (0.55)	1.51 (0.49)	1.46 (0.63)	1.38 (0.53)
	Venting	2.48 (0.76)	2.40 (0.76)	2.57 (0.83)	2.46 (0.69)
	Positive reframing	2.83 (0.71)	2.81 (0.67)	2.85 (0.77)	2.85 (0.69)
	Planning	3.19 (0.68)	3.09 (0.71)	3.21 (0.74)	3.26 (0.60)
	Humor	1.90 (0.71)	1.82 (0.69)	2.04 (0.82)	1.84 (0.58)
	Acceptance	3.03 (0.65)	3.01 (0.61)	2.96 (0.69)	3.12 (0.64)
	Religion	1.99 (0.99)	1.99 (1.00)	2.06 (1.02)	1.90 (0.94)
	Self-blaming	2.50 (0.74)	2.38 (0.70)	2.56 (0.66)	2.57 (0.84)
**STAI**	State scale	37.65 (9.65)	37.84 (9.93)	37.51 (9.24)	37.60 (9.90)
	Trait scale	36.37 (8.53)	35.20 (7.62)	35.87 (7.97)	38.06 (9.72)

Mean (and SD) are reported for age in years and for Brief-COPE and STAI scales. Number of observation (and percentages) are reported for the categorical measures. *Brief-COPE* Brief version of the Coping Orientation to the Problems Experienced; *STAI* Y form of the State-Tait Anxiety Inventory. * No data were available for one participant in the 2nd trimester group

### Measures

The socio-demographic data were collected with a patient form, including information on age, education, employment status, marital status, number of previous pregnancies, week of pregnancy, and patient medical history. Four psychometric instruments were used.

#### NuPCI (Revised Prenatal Coping Inventory)

The Italian translation of the revised version of the Prenatal Coping Inventory was used [10, 11]. Two of the authors (MB and CP) translated the original NuPCI in Italian, with the Authors’ permission, using the forward-backward procedure: one researcher translated the items in Italian and the second back-translated them. After performing the translations, the authors revised the Italian version in order to have a final instrument as similar as possible to the original NuPCI, but also adapted to the Italian cultural contest. Both translators are native Italian speakers fluent in English.

The questionnaire includes 42 items, rated on a five-point scale from zero (“Never”) to four (“Very often”). According to the factors reported by Hamilton and Lobel [11], three scales were considered: Planning-Preparation (PP; that includes 15 items: 1, 2, 3, 5, 11, 12, 13, 14, 17, 19, 23, 24, 34, 39, and 42), Avoidance (A; 11 items: 4, 7, 10, 18, 20, 26, 27, 30, 31, 37, and 38) and Spiritual-Positive Coping (SPC; 6 items: 6, 9, 16, 33, 36, and 41); higher scale scores correspond to a more frequent use of the specific coping style. In this English version the Planning-Preparation scale showed a Cronbach’s alpha of 0.82, 0.85 and 0.86 in early, mid-, and late pregnancy, respectively. The Avoidance scale showed a Cronbach’s alpha of 0.77, 0.79, and 0.80. Lastly, the Spiritual-Positive scale showed a Cronbach’s alpha of 0.73, 0.78, and 0.77 [11]. Moreover, the NuPCI subscales showed good validity, in association with a measure of coping strategies, the Way of Coping Questionnaire (WCQ): Planning-Preparation scale was associated with the WCQ Problem-Solving scale (*r* = + 0.542, *p* < 0.01), Avoidance scale was associated with Emotional-Solving scale (*r* = + 0.606, *p* < 0.01), and Spiritual-Positive Coping scale was associated with Problem-Solving scale (*r* = + 0.231, *p* < 0.01).

The Italian version of NuPCI used in this study can be requested by contacting the corresponding author.

#### NuPDQ (Revised Prenatal Distress Questionnaire)

The NuPDQ is the revised version of the Prenatal Distress Questionnaire [10], developed by Lobel et al. [7]. Initially, NuPDQ had three different forms (i.e., for early-, mid- and late-pregnancy), and it was subsequently modified to include all the items regardless of the pregnancy period [16].

It includes 18-items evaluating the level of PSS. The first 17 items are rated on a three-point scale from zero (“Never”) to two (“Very often”). The last item is rated zero-one (“Yes”/“No”). The NuPDQ provides a prenatal stress score ranging from zero to 35 (i.e., the sum of the item scores). This instrument showed good reliability (Cronbach’s coefficient alpha 0.55–0.79) and validity, considering the association with other instruments [18].

For this study, the NuPDQ was translated to Italian using the same forward-backward procedure adopted for the NuPCI. The Authors’ permission was also obtained.

The Italian version of NuPDQ used in this study can be requested by contacting the corresponding author.

#### Brief-COPE (Coping Orientation to the Problems Experienced)

The Brief-COPE is a 28-item self-report questionnaire designed to measure effective and ineffective ways of coping with stressful life events [4]. It is a short self-report instrument that showed good validity and reliability worldwide; it was translated in several languages, including Italian [29]. The original instrument includes 14 subscales, that showed a good reliability (subscales Cronbach’s alpha range between 0.50 and 0.90) [4]: Self-distraction, Active coping, Denial, Substance use, Emotional support, Use of instrumental support, Behavioral disengagement, Venting, Positive reframing, Planning, Humor, Acceptance, Religion, and Self-blame. According to previous research [9], the scores may also be summarized by two global coping styles: Avoidant Coping (derived by the subscales of Denial, Substance use, Venting, Behavioral disengagement, Self-distraction, and Self-blame) and Approach Coping (Active coping, Positive reframing, Planning, Acceptance, Seeking emotional support, and Seeking informational support). Compared to Approach Coping, Avoidant Coping is shown to be less effective at managing anxiety [30]. Moreover, other different measurement models have been proposed. Cooper and collaborators [31] identified three general coping styles (Problem-focused, Emotion-focused, and Dysfunctional coping) while Meyer [32] grouped the 14 original subscales in two styles: Adaptive and Maladaptive coping (see also: [33]). We have adopted this latter partition.

#### STAI (State-Trait Anxiety Inventory, Y form)

The STAI [34] comprises separate self-report scales for measuring state and trait anxiety. The State anxiety scale (STAI, Form Y-1) consists of 20 statements that evaluate how respondents feel “right now, at this moment”. The Trait anxiety scale (STAI, Form Y-2) consists of 20 statements that assess how people generally feel. It is a well validated instrument (the Cronbach’s alpha coefficients for trait and state anxiety scales revealed a high reliability in female adults, 0.91 and 0.93 respectively) [34], used worldwide. We used the Italian version of the STAI [35].

### Data analysis

Continuous measures were summarized using means and standard deviation (SD). Between-group differences were analyzed using analysis of variance, or Kruskal-Wallis’ test, when the assumption of homogeneity of variance was violated. For statistically significant results post-hoc analyses were conducted using Tukey’s honest significance test. Partial omega-square (ω^2^_P_) was used for effect-size estimations, conventionally considering effects as medium-size when ω^2^_P_ was between 0.06 and 0.15. Pearson’s product-moment correlation coefficient (r) was also calculated.

For categorical measures, between-group comparisons were performed using χ^2^-test.

Shapiro-Wilk’s test was used to evaluate normality of distribution of NuPCI scales and NuPDQ scores.

The internal consistency of NuPCI scales was assessed using Cronbach’s coefficient alpha (ɑ_C_). Internal consistency is good when ɑ_C_ ≥0.8 and acceptable when ɑ_C_ ≥0.6. Also, maximum-likelihood exploratory factor analyses (EFAs) were conducted for each scale. Unidimensionality was evaluated as the proportion of variance explained by the 1st factor and the ratio between 1st and 2nd eigenvalue extracted (i.e., expected to be > 3). Confirmatory factor analyses (CFAs) were conducted first testing the original NuPCI measurement model (i.e., with 32 items organized on 3 orthogonal scales). The measurement model was then modified excluding three items and allowing correlated factors. The diagonally weighted least squares estimator, suitable for five-levels ordinal items, were used and robust test was preferred. A model fit was considered acceptable for a ratio between model’s χ^2^ and degrees of freedom less than two, a Comparative Fit Index (CFI) and Tucker-Lewis’ Index (TLI) greater than 0.900, and a Root Mean Square Error of Approximation (RMSEA) less than 0.050.

To investigate the effects of NuPCI scales on the NuPDQ score, a multiple linear regression was fitted.

To check the assumption for the use of multiple linear regression, Mardia’s multivariate skewness and kurtosis coefficients were calculated and Breusch-Pagan’s test for multivariate heteroskedasticity was used.

Variance inflation factors were calculated, considering acceptable values below two. The ability of coping strategies (NuPCI scales) to predict Apgar score was estimated in by-trimester samples. Multiple linear regression models with covariates only (i.e., week of pregnancy, age of participant, status of primigravida, and presence of previous miscarriage) were compared with models including also NuPCI scales (best-fit χ^2^-test). Then, NuPDQ scores were introduced in the selected models, evaluating their possible moderating effect. We excluded from those analyses two women who lost their baby before birth and three participants with twin-delivery.

The 95% confidence intervals used to evaluate the statistical significance of item-loadings in EFAs and those reported for ɑ_C_ and ω^2^_P_ were calculated with a bootstrapping procedure (using 10,000 replication samples).

The level of statistical significance was set at ɑ=0.05. Since a total of 22 independent scales were included (i.e., three from NuPCI, NuPDQ, 16 from Brief-COPE, and two from STAI), in correlation analysis statistical significance was considered for *p* ≤ 0.002, possibly indicating as close to statistical significance those correlations that did not survive to this correction.

All analyses were conducted using R-3.6.3 [36], using Lavaan 0.6-6 library in conducting CFAs [37].

## Results

### Socio-demographic characteristics

The age of participants ranged from 20 to 46 years (mean: 32.91 years, SD: 4.95). About half of women (49.8%) were at their first pregnancy, 30.8% was at their second one, and 19.4% had two or more previous pregnancies. About half of the sample (47.9%) had a university education, while 42.2% had finished high school; 9.5% had a lower education (primary and middle school). In the total sample, about one quarter was unemployed, 65.4% was employed, and 9.5% was self-employed. As for the marital status, 64.9% of women were married, 34.1% single and 0.9% divorced.

No statistically significant differences were found on age (F_2,208_=0.30, *p* = 0.744), previous pregnancy (any previous pregnancy: χ^2^_2_ = 0.63, *p* = 0.729; number of pregnancy up to two: χ^2^_4_ = 6.20, *p* = 0.184), education (three levels: χ^2^_4_ = 1.79, p = 0.775), employment (χ^2^_4_ = 3.31, *p* = 0.508) and marital (excluding divorced: χ^2^_2_ = 0.13, *p* = 0.936) status between pregnant women in the first, second and third trimester. Similarly, no between-group difference resulted in Brief-COPE (all with *p* ≥ 0.132) and STAI (all with *p* ≥ 0.117) scales. Socio-demographic characteristics, Brief-COPE and STAI scores are reported in Table 1. Scores of the NuPCI scale are reported in Table 2.

**Table 2:** Mean and Cronbach’s coefficient alphas

**Trimester**	**Sample**	**PP**	**A**	**SPC**
**1st**	North American	2.05 (0.68) ɑ_C_=0.82	1.34 (0.69) ɑ_C_=0.77	2.41 (0.80) ɑ_C_=0.73
	Italian	1.79 (0.734) ɑ_C_=0.87 [0.82–0.91]	0.88 (0.510) ɑ_C_=0.69 [0.55–0.78]	1.66 (0.962) ɑ_C_=0.83 [0.77–0.84]
**2nd**	North American	2.09 (0.70) ɑ_C_=0.85	1.28 (0.65) ɑ_C_=0.79	2.36 (0.82) ɑ_C_=0.78
	Italian	2.05 (0.550) ɑ_C_=0.75 [0.63–0.82]	0.97 (0.653) ɑ_C_=0.79 [0.70–0.84]	1.69 (0.977) ɑ_C_=0.82 [0.75–0.86]
**3rd**	North American	2.05 (0.72) ɑ_C_=0.86	1.26 (0.65) ɑ_C_=0.80	2.25 (0.83) ɑ_C_=0.77
	Italian	2.29 (0.624) ɑ_C_=0.82 [0.75–0.87]	1.00 (0.595) ɑ_C_=0.76 [0.67–0.83]	1.76 (0.958) ɑ_C_=0.79 [0.72–0.84]
**Total sample**	Italian	2.04 (0.669) ɑ_C_=0.84 [0.80–0.87]	0.95 (0.589) ɑ_C_=0.76 [0.70–0.80]	1.70 (0.962) ɑ_C_=0.81 [0.77–0.84]

Mean (SD) and Cronbach’s coefficient alphas (with 95% confidence interval) for Revised Prenatal Coping Inventory (NuPCI) scales in North American [11] and Italian samples. *A* NuPCI, Avoidance scale; *PP* NuPCI, Planning-Preparation scale; *SPC* NuPCI, Spiritual-Positive Coping scale

The PP scale scores had a normal distribution in all three trimester groups (respectively: W = 0.98, *p* = 0.182; W = 0.984, *p* = 0.493; and W = 0.99, *p* = 0.657), while the A and SPC scales had a normal distribution in 3rd trimester (A: W = 0.97, *p* = 0.115; and SPC: W = 0.97, *p* = 0.124) and a non-normal one in 1st (W = 0.94, *p* = 0.004; W = 0.95, *p* = 0.004) and 2nd (W = 0.96, *p* = 0.017; W = 0.94, *p* = 0.003) trimesters.

**Fig. 1:**
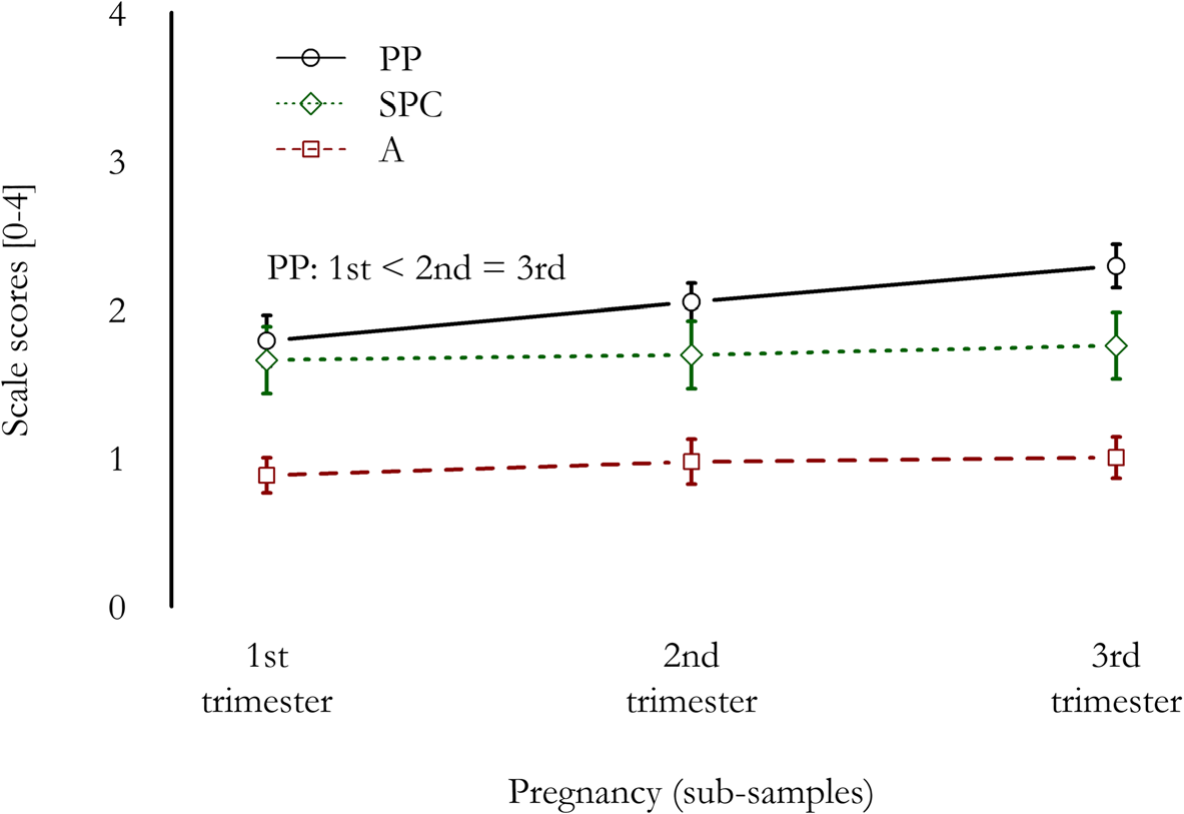
NuPCI scale scores in the three trimesters. Mean scores and their 95% confidence intervals are reported. *A*: NuPCI, Avoidance scale; *PP*: NuPCI, Planning-Preparation scale; *SPC*: NuPCI, Spiritual-Positive Coping scale

The trimester of pregnancy showed a statistical significant effect for PP scale (F_2,208_=10.81, *p* < 0.001; medium-size effect: ω^2^_P_ = 0.085 [0.011, 0.176]), with lower score in 1st trimester group than in 2nd (post-hoc test: *p* = 0.045) and 3rd (*p* < 0.001) ones, and no statistically significant difference between them (*p* = 0.065). An increasing trend in scores was observed from 1st to 3rd trimester (see Fig. 1). No significant differences among trimesters were found for A and SPC scales (respectively, F_2,208_=0.79, *p* = 0.456; and F_2,208_=0.18, *p* = 0.832), or for NuPDQ score (F_2,208_=1.65, *p* = 0.195).

### Internal consistency

Considering the item organization proposed by Hamilton and Lobel (2008), in the total sample, good internal consistency was observed for PP (15 items; ɑ_C_=0.84 [0.81–0.87]) and SPC (6 items; ɑ_C_=0.81 [0.77–0.84]) scales. A lower, but acceptable, result was obtained for A scale (11 items; ɑ_C_=0.76 [0.70–0.80]). Acceptable-to-good internal consistencies were found in trimester samples (ɑ_C_ ranging from 0.69 to 0.87; see Table 2). In EFA, a single factor explains the 27.8% of the variance of the items included in the PP scale, with a ratio between 1st and 2nd eigenvalue of 6.3. Similarly, a unidimensional solution resulted acceptable also for items of A scale (23.7%; ratio of 4.8) and of SPC one (45.2%; ratio of 6.1). All item loadings in scale-by-scale EFAs were statistically significant. Low loadings (< 0.3) were observed for item 39 (PP: +0.191, *p* = 0.014), item 7 (A: +0.228, *p* = 0.013), and item 9 (SPC: +0.241, *p* = 0.001).

### Construct validity

In CFA, the NuPCI original measurement model did not adequately fit current data: χ^2^_464_ = 1282.03 (*p* < 0.001); χ^2^_464_/df = 2.76; RMSEA = 0.092 (90% ci: [0.086, 0.098], p_(RMSEA≤0.050)_ < 0.001); CFI = 0.738; and TLI = 0.720. In particular, it seemed not to account for correlations between scales scores. Refining the model, we allowed the correlation between factors and excluded three items (i.e., number 7 on scale A; 39 on PP; and 9 on SPC), obtaining a model with adequate fit: χ^2^_374_ = 618.06; χ^2^_374_/df = 1.65; RMSEA = 0.056 (90% ci: [0.048, 0.063], p_(RMSEA≤0.050)_ = 0.113); CFI = 0.920; and TLI = 0.913.

Considering the excluded items, the residuals of item 7 (i.e., *“Tried to keep your feelings about being pregnant to yourself?”*) were correlated with those of other two items with similar content (both on scale A): *“Tried to keep your feelings about the pregnancy from interfering with things you had to do?”* and *“Tried to stay away from other people?”*. Resulting in a non-statistically significant loading of the corresponding factor. Item 39 (*“Felt that having a baby was fulfilling a lifetime dream or goal?”*) showed to be associated with both PP factor and SPC one. Examining its content, it could probably be interpreted focusing on “lifetime goal” (possibly more associated with current PP scale) or on “lifetime dream” (possibly more associated with SPC scale). Finally, item 9 (*“Tried to focus on what is important in life?”*) was similarly associated with scale SPC and to scale PP. As for item 39, its content could possibly be interpreted both as: (i) to focus on important life values or spiritual aspects (i.e., in line with current SPC scale), and (ii) to focus on important current plans or practical aspects (i.e., in line with PP scale).

In the final model, all the item loadings were statistically significant with respect to the corresponding factor (see Table 3). Factor PP was positively correlated with factor A (estimated in: +0.481, *p* < 0.001) and factor SPC (+ 0.186, *p* = 0.005), and A and SPC were also positively correlated (+ 0.349, *p* < 0.001).

**Table 3:** Item loadings from confirmatory factor analysis (29 items)

**NuPCI scale**	**Item**	**Estimate (se)**	**z-value**
PP	01	+ 0.614 (0.046)	+ 13.332
	02	+ 0.639 (0.044)	+ 14.571
	03	+ 0.440 (0.059)	+ 7.452
	05	+ 0.522 (0.053)	+ 9.915
	11	+ 0.630 (0.046)	+ 13.799
	12	+ 0.663 (0.045)	+ 14.831
	13	+ 0.547 (0.045)	+ 12.147
	14	+ 0.523 (0.059)	+ 8.886
	17	+ 0.546 (0.055)	+ 9.904
	19	+ 0.496 (0.052)	+ 9.518
	23	+ 0.541 (0.053)	+ 10.292
	24	+ 0.480 (0.057)	+ 8.467
	34	+ 0.846 (0.028)	+ 29.744
	42	+ 0.645 (0.045)	+ 14.262
A	04	+ 0.513 (0.064)	+ 8.042
	10	+ 0.629 (0.058)	+ 10.793
	18	+ 0.321 (0.068)	+ 4.691
	20	+ 0.721 (0.051)	+ 14.077
	26	+ 0.761 (0.049)	+ 15.632
	27	+ 0.534 (0.063)	+ 8.460
	30	+ 0.745 (0.053)	+ 13.925
	31	+ 0.514 (0.063)	+ 8.185
	37	+ 0.519 (0.067)	+ 7.710
	38	+ 0.406 (0.067)	+ 6.058
SPC	06	+ 0.840 (0.045)	+ 18.548
	16	+ 0.859 (0.027)	+ 31.412
	33	+ 0.936 (0.027)	+ 34.626
	36	+ 0.830 (0.038)	+ 22.002
	41	+ 0.766 (0.042)	+ 18.431

*A* NuPCI, Avoidance scale; *PP* NuPCI, Planning-Preparation scale; *se* standard error; *SPC* NuPCI, Spiritual-Positive Coping scale

### Convergent validity

Correlations between NuPCI scales and the subscales of Brief-COPE are shown in Table 4. After correction for multiple comparisons, statistical significance was set to *p* ≤ 0.002. A trend to statistical significance at *p* < 0.050 was also defined. The PP score was positively correlated with the Self-Distraction (*r* = + 0.217, *p* = 0.001) Brief-COPE subscale. A positive trend to statistical significance was found for both Adaptive and Maladaptive Coping, as well as for the following subscales: Use of emotional support, Use of instrumental support and Positive reframing. The A score significantly correlated with Maladaptive Coping (*r* = + 0.387, *p* < 0.001) and with the subscales Denial (*r* = + 0.238, *p* < 0.001), Use of emotional support (*r* = + 0.259, *p* < 0.001), Use of instrumental support (*r* = + 0.219, *p* = 0.001), Behavioral disengagement (*r *= + 0.218, *p* = 0.001), and Self-blaming (*r* = + 0.289, *p* < 0.001). Trends to statistical significance were found for Self-Distraction and Venting subscales. The SPC scale significantly correlated with Adaptive coping (*r* = + 0.292, *p* < 0.001) and with Religion (*r* = + 0.624, *p* < 0.001) Brief-COPE subscale. Denial and Positive reframing showed a positive trend to statistical significance.

**Table 4:** Convergent validity

**Scale**	**PP**	**A**	**SPC**	**NuPDQ**
**Brief-COPE**				
Adaptive coping	+ 0.165 (0.016)	+ 0.117 (0.091)	+ 0.292 (< 0.001)*	-0.034 (0.619)
Maladaptive coping	+ 0.188 (0.006)	+ 0.387 (< 0.001)*	+ 0.079 (0.254)	+ 0.263 (< 0.001)*
Self-distraction	+ 0.217 (0.001)*	+ 0.181 (0.008)	+ 0.117 (0.089)	+ 0.110 (0.111)
Active coping	-0.045 (0.517)	-0.053 (0.446)	-0.034 (0.625)	-0.068 (0.328)
Denial	+ 0.102 (0.141)	+ 0.238 (< 0.001)*	+ 0.170 (0.013)	+ 0.121 (0.079)
Substance use	-0.025 (0.717)	+ 0.106 (0.126)	-0.103 (0.138)	+ 0.046 (0.503)
Use of emotional support	+ 0.189 (0.006)	+ 0.259 (< 0.001)*	+ 0.078 (0.258)	+ 0.236 (0.001)*
Use of instrumental support	+ 0.162 (0.019)	+ 0.219 (0.001)*	+ 0.127 (0.066)	+ 0.155 (0.024)
Behavioral disengagement	+ 0.045 (0.516)	+ 0.218 (0.001)*	+ 0.088 (0.202)	+ 0.196 (0.004)
Venting	+ 0.085 (0.218)	+ 0.162 (0.018)	-0.021 (0.761)	+ 0.172 (0.012)
Positive reframing	+ 0.205 (0.003)	+ 0.099 (0.152)	+ 0.196 (0.004)	-0.059 (0.398)
Planning	+ 0.079 (0.253)	+ 0.011 (0.873)	+ 0.004 (0.949)	-0.072 (0.295)
Humor	+ 0.083 (0.229)	+ 0.074 (0.283)	+ 0.062 (0.368)	-0.053 (0.448)
Acceptance	-0.003 (0.960)	-0.110 (0.111)	+ 0.111 (0.107)	-0.161 (0.019)
Religion	+ 0.071 (0.302)	+ 0.005 (0.941)	+ 0.624 (< 0.001)*	-0.151 (0.028)
Self-blaming	+ 0.085 (0.220)	+ 0.289 (< 0.001)*	-0.047 (0.496)	+ 0.147 (0.033)
**STAI-Y**				
State scale	+ 0.149 (0.031)	+ 0.547 (< 0.001)*	+ 0.042 (0.544)	+ 0.539 (< 0.001)*
Trait scale	+ 0.062 (0.367)	+ 0.466 (< 0.001)*	+ 0.016 (0.814)	+ 0.462 (< 0.001)*

Correlations (p) between NuPCI scales and Brief-COPE subscale and between NuPDQ scale and STAI (State and Trait Scales). *A* NuPCI, Avoidance scale; *PP* NuPCI, Planning-Preparation scale; *SPC* NuPCI, Spiritual-Positive Coping scale; *STAI-Y* Y form of the State-Trait Anxiety Inventory. * Statistically significant effects after correction for multiple independent comparisons (*p* ≤ 0.002)

### Concurrent validity

No violation of the assumptions underlying multiple linear regression was detected.

**Fig. 2:**
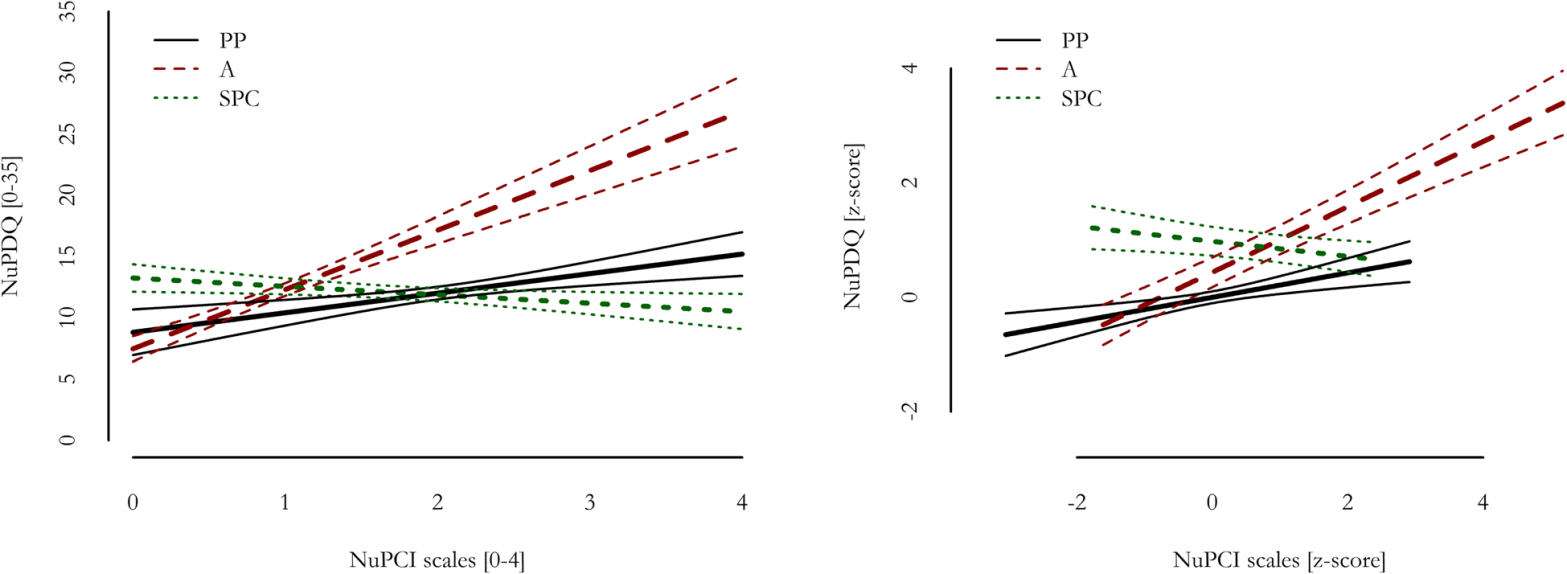
Relationship between NuPDQ and NuPCI scales, with 95% confidence intervals (multiple linear regression model). Scale scores are reported on the left, scale z-scores based on this sample on the right. All predictors were statistically significant (with *p* ≤ 0.022). *A*: NuPCI, Avoidance scale; *PP*: NuPCI, Planning-Preparation scale; *SPC*: NuPCI, Spiritual-Positive Coping scale

**Fig. 3:**
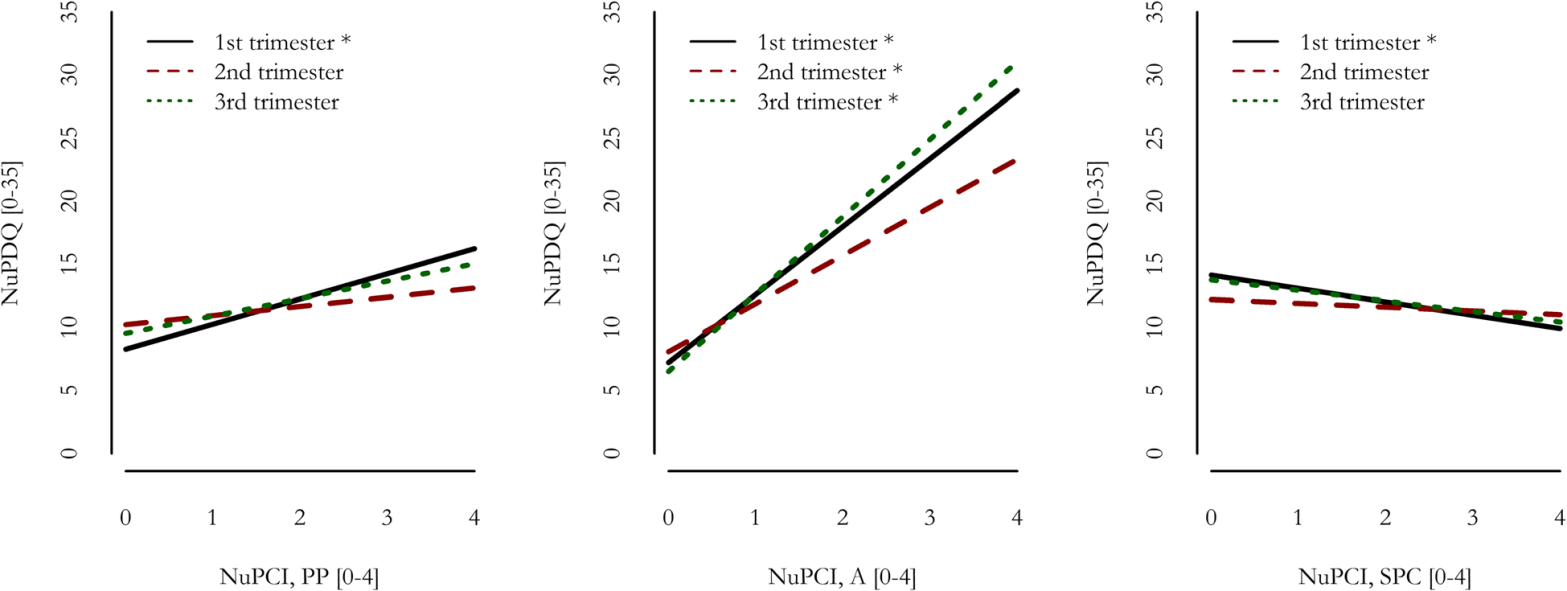
Results of multiple linear regression models fitted by-trimester. NuPDQ predicted by NuPCI scales (Planning-Preparation, Avoidance and Spiritual-Positive Coping). *A*: NuPCI, Avoidance scale; *PP*: NuPCI, Planning-Preparation scale; *SPC*: NuPCI, Spiritual-Positive Coping scale. *: Statistically significant predictor

Figure 2 Table 5 show the effects of NuPCI scales on NuPDQ. To note, NuPDQ score was positively correlated with STAI scales (Table 4), showing a good validity in the assessment of stress. NuPDQ score was predicted by all NuPCI scales (R^2^ = 0.423, F_3,207_=50.55, *p* < 0.001). The score was positively predicted by A (B = + 4.85, *p* < 0.001; β=+0.572) and PP (B = + 1.60, *p* < 0.001; β=+0.215) scores. On the contrary, the SPC score was negatively associated with NuPDQ (B=-0.69, p = 0.022; β=-0.132). Results showed were in the same direction when separate by-trimester samples were considered, with statistically significant effects of A scores in all sub-samples and of PP and SPC in the 1st trimester only (see Table 5; Fig. 3).

**Table 5:** Relationship between NuPDQ and NuPCI scales. Results of multiple linear regression models fitted by-trimester

	**Predicted: NuPDQ**			**PP**		**A**		**SPC**	
**Trimester**	**Df**	**F (p)**	**R**^**2**^	**B (p)**	**Β**	**B (p)**	**β**	**B (p)**	**Β**
**1st**	3 / 66	17.22 (< 0.001)	0.438	+ 1.99 (0.004)	+ 0.308	+ 5.39 (< 0.001)	+ 0.580	-1.05 (0.043)	-0.214
**2nd**	3 / 67	9.39 (< 0.001)	0.296	+ 0.72 (0.474)	+ 0.083	+ 3.81 (< 0.001)	+ 0.521	-0.30 (0.588)	-0.060
**3rd**	3 / 66	26.73 (< 0.001)	0.549	+ 1.37 (0.113)	+ 0.159	+ 6.13 (< 0.001)	+ 0.676	-0.83 (0.098)	-0.147
**Total sample**	3 / 207	50.55 (< 0.001)	0.423	+ 1.60 (< 0.001)	+ 0.215	+ 4.85 (< 0.001)	+ 0.572	-0.69 (0.022)	-0.132

*A* NuPCI, Avoidance scale; *B* Regression coefficient; *β* Standard regression coefficient; *Df* Degrees of freedom of the model; *PP* NuPCI, Planning-Preparation scale; *R*^*2*^ Coefficient of multiple determination of the model; *SPC* NuPCI, Spiritual-Positive Coping scale

### Predictive validity

**Fig. 4:**
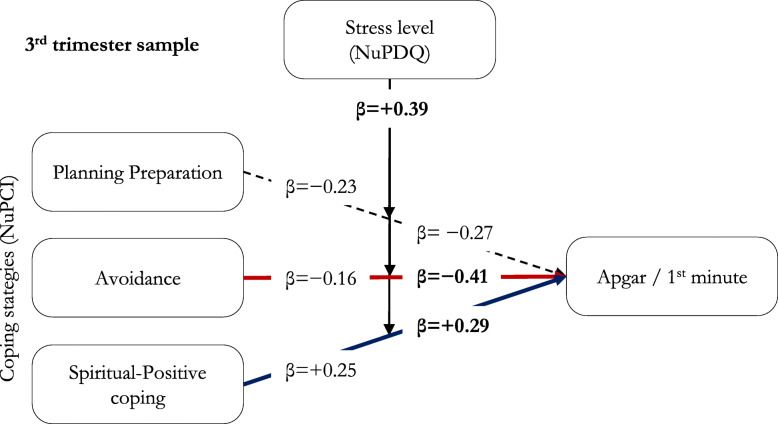
Moderation effects from multiple linear regression model. Apgar score at one minute predicted by coping strategies (NuPCI scales), with the moderation of the level of stress (NuPDQ score). The model for the 3rd trimester sample is represented. Solid lines represent statistically significant predictors; dashed lines represent not statistically significant predictors. Statistically significant β are reported in bold

**Fig. 5:**
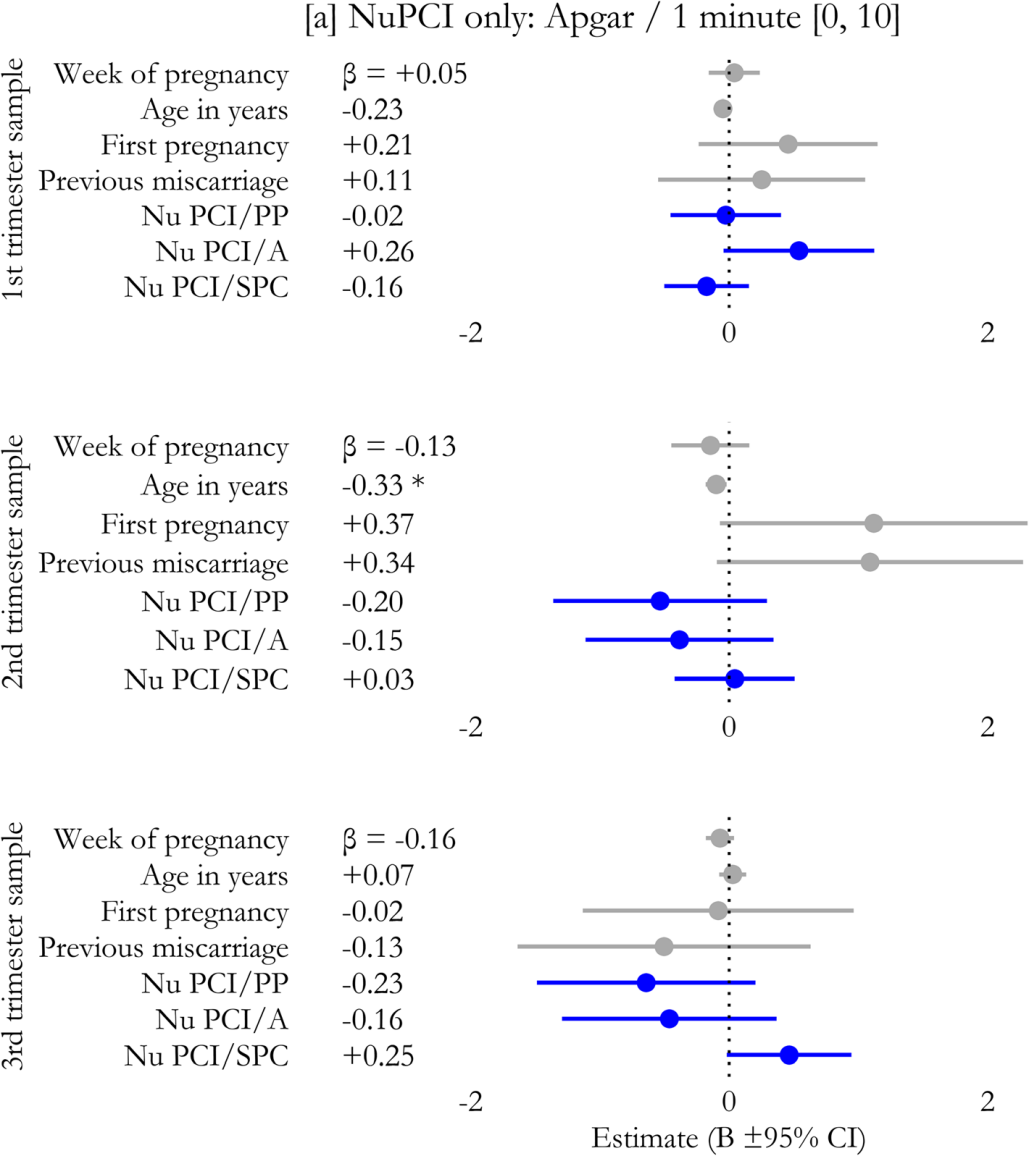
Prediction of the Apgar score at one minute by coping strategies only (NuPCI scales). Week of pregnancy, age of participant, status of primigravida, and presence of previous miscarriage are included in the models as covariates. Only the model for 3rd trimester sample statistically significantly improved when stress level was included. Estimated coefficients (B) and corresponding 95% confidence intervals are plotted; standardized coefficients values (β) are reported. Colors were used to mark the different categories of predictors. *A*: NuPCI, Avoidance scale; *PP*: NuPCI, Planning-Preparation scale; *SPC*: NuPCI, Spiritual-Positive Coping scale. *: Statistically significant with *p *< 0.050 (t-test)

**Fig. 6:**
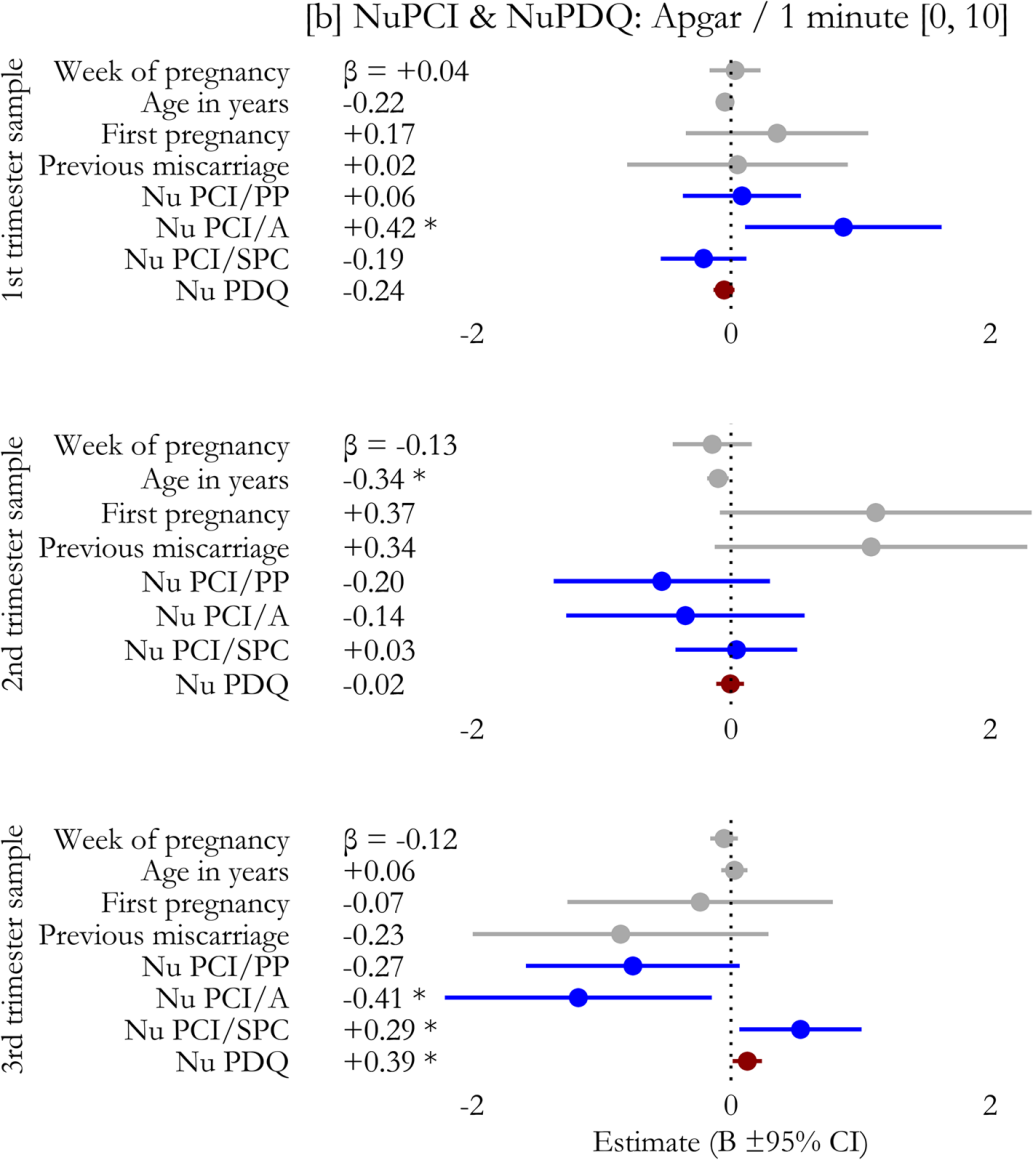
Prediction of the Apgar score at one minute including also stress level (NuPDQ score). Week of pregnancy, age of participant, status of primigravida, and presence of previous miscarriage are included in the models as covariates. Only the model for 3rd trimester sample statistically significantly improved when stress level was included. Estimated coefficients (B) and corresponding 95% confidence intervals are plotted; standardized coefficients values (β) are reported. Colors were used to mark the different categories of predictors. *A*: NuPCI, Avoidance scale; *PP*: NuPCI, Planning-Preparation scale; *SPC*: NuPCI, Spiritual-Positive Coping scale. *: Statistically significant with *p* < 0.050 (t-test)

In regression models, no significant effect on 1-minute Apgar scores was observed including only the covariates (1st trimester: χ^2^_4_ = 5.49, *p* = 0.282; R^2^ = 0.079; 2nd : χ^2^_4_ = 14.93, *p* = 0.153; R^2^ = 0.111; 3rd : χ^2^_4_ = 12.70, *p* = 0.282; R^2^ = 0.067). The introduction of NuPCI scale improved the model in the 3rd trimester sample (χ^2^_3_ = 20.50, *p* = 0.043; R^2^ = 0.176, + 161.4%), but not in the 1st (χ^2^_3_ = 4.84, *p* = 0.216; R^2^ = 0.148) and 2nd (χ^2^_3_ = 8.54, *p* = 0.280; R^2^ = 0.174) trimester samples. The addition of the NuPDQ scale further improved the model in 3rd trimester (χ^2^_1_ = 12.43, *p* = 0.026; R^2^ = 0.241, + 37.4%), making significant the effects of A (B=-1.179, β=-0.408; t=-2.29, *p* = 0.026) and SPC (B = + 0.537, β=+0.293; t = + 2.28, *p* = 0.026) scales, as well as that of NuPDQ score (B = + 0.126, β=+0.389; t = + 2.22, *p* = 0.030; Fig. 4). This means that when the stress was considered, the effect of the Avoidance score in decreasing 1-minute Apgar score became stronger (+ 155%), as well as that of Spiritual-Positive Coping in increasing 1-minute Apgar score (+ 16%). See also Figs. 5 and 6.

## Discussion

The main goal of this study was to analyze the psychometric properties of the Italian version of NuPCI in three groups of women assessed in different trimesters of their pregnancy.

In the literature, most of the studies considered coping styles in high-risk or complicated pregnancies [38], even if they represent a low percentage of the total gestations (i.e., about 12% of the pregnancy in the Italian population) [39].

This study focused on low-risk pregnant women to assess stress expression and coping strategies in typical situations. Furthermore, the sample included women in different trimesters of pregnancy. Many studies had focused on specific periods of pregnancy and only a few had examined coping across all pregnancy [7]. This is an important point because it is well known that stressors undergo main changes during pregnancy, probably with concomitant modifications in the coping strategies (e.g., [16]).

In our sample, no statistically significant heterogeneity was found for socio-demographic characteristics, general coping strategies (Brief-COPE), and levels of general (STAI) and pregnancy-specific (NuPDQ) stress among the three trimesters. Thus, the comparison between by-trimester samples in terms of coping strategies is reliable.

### Internal consistency

The Italian version of NuPCI showed good internal consistency (Cronbach’s coefficient alpha) for Planning-Preparation and Spiritual-Positive Coping scales and an acceptable level for Avoidance scale. These results, obtained both for the total sample and for the single trimesters, are in line with those reported for the North-American version of NuPCI [11] and with the outcomes of different validation studies conducted worldwide (i.e., for NuPCI Spanish and Persian versions) [12, 14].

The exploratory factor analyses conducted for each scale on the corresponding items showed that the scales were one-dimensional, although with poor results for three items (i.e., one for each scale).

### Construct validity

The measurement model proposed by Hamilton and Lobel [11] was not suitable for the present sample. However, a slightly adjusted model showed to fit adequately our data. Differently from the results of the original instruments, we observed correlated scale scores. In particular, the Planning-Preparation scale was weakly positively correlated with both Spiritual-Positive Coping and Avoidance, and a trend towards a significant positive correlation was found between the latter two scales. Also, three items were excluded from the final model, apparently because of redundancy (i.e., for item-7) and because of a possible ambiguous interpretation of their content (i.e., item-39, item-9). In view of the small differences observed and taking into account the size of our sample, we preferred to maintain the standard scoring method, to improve comparability with the original instrument.

### Convergent validity of coping scales

Planning-Preparation was the most used coping style (the average of the total sample is in “Sometimes” range), a result in line with Lorén-Guerrero’s work [14] but different from North-American results [11], in which women showed to use mainly Spiritual-Positive Coping. These discordant results could be explained by cultural differences, but also by differences in samples: low-risk pregnancies were considered in our and the Spanish studies, while Hamilton and Lobel’s work included also high-risk women. Besides, this scale significantly increased comparing the first trimester to the second and third ones, differently from the North-American sample [11], in which Planning-Preparation was stably used across all the three trimesters. However, in our study we reported cross-sectional observations, while in the original work the same women were longitudinally followed across the entire gestation. Planning-Preparation scale was positively correlated to the Self-Distraction Brief-COPE subscale. In addition, a positive trend to statistical significance was found for both Adaptive and Maladaptive Brief-COPE categories and for Use of emotional support, Use of instrumental support and Positive reframing subscales.

Spiritual-Positive coping style was used with a lower frequency than in the original sample (i.e., between “Rarely” and “Sometimes”) and with no differences between the trimesters. The Spiritual-Positive Coping scale was correlated with the Adaptive Coping scale and with Religion Brief-COPE subscale. These results confirmed that the scale is a direct measure of adaptive coping strategies.

Avoidance coping was the least common employed strategy, a result in line with many previous studies [11, 14]. As expected, the Avoidance scale significantly correlated with the Maladaptive Brief-COPE category and with Denial, Use of emotional support, Use of instrumental support, Behavioral disengagement and Self-blaming subscales. Also, trends to statistical significance were found for Self-Distraction and Venting subscales.

### Concurrent validity

We analyzed how coping strategies measured with NuPCI could predict PSS, as measured with NuPDQ. In our sample, NuPDQ demonstrated a good construct validity, since the total score was correlated with STAI State and Trait scales. In particular, there was a strong correlation with the State scale of STAI, which has often been used to assess PSS [40, 41]. All NuPCI scales were associated with NuPDQ score, but with different directions. Avoidance and Planning-Preparation scales positively predicted the NuPDQ score, with higher scores corresponding to greater perceived stress. Particularly, the Avoidance score showed a medium-size association with stress, so that the increase of one in this scale roughly corresponds to an increment of five in NuPDQ (or about half standard deviation for each standard deviation in predictor). According to a systematic review [1], avoidant approach affects mental health outcomes during pregnancy (and post-partum), increasing perceived stress, depression, anxiety, and subsequent child abuse. Moreover, avoidance coping has been associated with a higher level of corticotrophin-releasing hormone of placental origin (pCRH), which may lead to a preterm delivery [42, 43]. On the contrary, the Spiritual-Positive Coping scale negatively predicted the NuPDQ score (i.e., the higher was the score, the lower was the perceived stress). This finding is in line with the literature, where Spiritual approach had been linked with lower levels of stress in pregnant women without any risk [44]. Interestingly, in high-risk pregnant women, Spiritual-Positive Coping style has been instead associated with greater stress [45], suggesting that prayer could be seen as a form of rumination, rather than a reaction that gives relief [19].

### Predictive validity

The coping strategies showed significant effects in predicting Apgar score in the 3rd trimester sample, so that the linear model explained 17% of its variance (where the covariates only explained the 7% of it). Furthermore, in this group, the level of pregnancy-specific stress assessed with PDQ moderated the predictions of coping strategies (i.e., with the 24% of explained variance). Considering the moderating level of stress, a mother’s Avoidance style in the third trimester was associated with a worsening of the baby’s ability to tolerate the birth process, while a Spiritual-Positive coping was associated with an improvement in this capacity. Inconsistent results have been previously reported considering the association of stress and Apgar score [24], recommending that birth status could be better predicted with mediating factors [46] or multiple moderators [23]. Our results may suggest a significant role of coping strategies, and in particular those close to the childbirth.

### Limitations

The main limitation of this work is the adopted cross-sectional design. Thus, it was not possible to examine the test-retest reliability of the Italian NuPCI and to follow the same women through pregnancy. Nevertheless, this was partially overcome by the homogeneity observed in the three trimesters. Another limitation is the relatively low sample size, even though it is in line with other validation studies [12, 14, 47]. Moreover, we performed only a forward- and back-translation for our Italian version, instead of following specific recommendations for cross-cultural adaptation (e.g., Beaton’s recommendation [48]). A further limitation could be that some of the correlations between NuPCI and Brief-COPE scales statistically significant at ≤ 0.002 were moderate in absolute terms. Finally, choosing to include only women with low-risk pregnancies does not allow to observe the burden of stress related to pathological conditions on pregnancy.

## Conclusions

This is the first study to assess the psychometric properties of NuPCI in an Italian sample. In our study, NuPCI demonstrated a good internal consistency and a three factor structure similar to the original version. The convergent validity analysis showed good correlations between NuPCI scales and Brief-COPE styles. Moreover, Avoidance and Planning-Preparation NuPCI scales predicted positively PSS, while Spiritual-Positive scale predicted it negatively. Finally, the predictive validity analysis suggests a clinical utility of NuPCI, as different coping styles in the third trimester of pregnancy have been associated with newborn conditions. Overall, NuPCI is valid and reliable in low-risk pregnant women. Moreover, also NuPDQ showed good validity, when compared with a general measure of stress.

## Data Availability

The datasets used and analyzed during the current study are available from the corresponding author on reasonable request.

## Abbreviations

A: NuPCI avoidance scale
ɑC: Cronbach's coefficient alpha
Brief-COPE: Coping orientation to the problems experienced
EFAs: Exploratory factor analyses
NuPCI: Revised prenatal coping inventory
NuPDQ: Revised-prenatal distress questionnaire
PP: NuPCI planning preparation scale
PSS: Pregnancy-specific stress
SD: Standard deviation
SPC: NuPCI spiritual-positive coping scale
STAI: State-trait anxiety inventory
